# *OPRM1* c.118A>G Polymorphism and Duration of Morphine Treatment Associated with Morphine Doses and Quality-of-Life in Palliative Cancer Pain Settings

**DOI:** 10.3390/ijms18040669

**Published:** 2017-03-27

**Authors:** Aline Hajj, Lucine Halepian, Nada El Osta, Georges Chahine, Joseph Kattan, Lydia Rabbaa Khabbaz

**Affiliations:** 1 Laboratory of Pharmacology, Clinical Pharmacy and Quality Control of Drugs, Pôle Technologie- Santé (PTS), Faculty of Pharmacy, Saint-Joseph University, Beirut 1107 2180, Lebanon; lucine.halepian@hotmail.com (L.H.); lydia.khabbaz@usj.edu.lb (L.R.K.); 2 Department of Public Health, Faculty of Medicine, Saint-Joseph University, Beirut 1107 2180, Lebanon; pronada99@hotmail.com; 3 Department of Prosthodontics, Faculty of Dental Medicine, Saint-Joseph University, Beirut 1107 2180, Lebanon; 4 University of Auvergne, CROC-EA4847, Centre de Recherche en Odontologie Clinique, BP 10448, Clermont-Ferrand F-63000, France; 5 Department of Hemato-Oncology, Hôtel-Dieu de France Hospital, Faculty of Medicine, Saint-Joseph University, Beirut 1107 2180, Lebanon; chahine_georges@hotmail.com (G.C.); joseph.kattan@usj.edu.lb (J.K.)

**Keywords:** morphine, polymorphism, *OPRM1*, *ABCB1*, *COMT*, pain, pharmacogenetics, cancer, quality-of-life

## Abstract

Despite increased attention on assessment and management, pain remains the most persistent symptom in patients with cancer, in particular in end-of-life settings, with detrimental impact on their quality-of-life (QOL). We conducted this study to evaluate the added value of determining some genetic and non-genetic factors to optimize cancer pain treatment. Eighty-nine patients were included in the study for the evaluation of palliative cancer pain management. The regression analysis showed that age, *OPRM1* single nucleotide polymorphism (SNP), as well as the duration of morphine treatment were significantly associated with morphine doses at 24 h (given by infusion pump; *p* = 0.043, 0.029, and <0.001, respectively). The mean doses of morphine decreased with age but increased with the duration of morphine treatment. In addition, patients with AG genotype c.118A>G *OPRM1* needed a higher dose of morphine than AA patients. Moreover, metastases, *OPRM1* SNP, age, and gender were significantly associated with the QOL in our population. In particular, AA patients for *OPRM1* SNP had significantly lower cognitive function than AG patients, a result not previously reported in the literature. These findings could help increase the effectiveness of morphine treatment and enhance the QOL of patients in regards to personalized medicine.

## 1. Introduction

Pain is the most persistent and common symptom in cancer patients, especially in the end-of-life setting of palliative care. Hence, international guidelines, such as those emitted during the latest American Society of Clinical Oncology (ASCO) meeting, health care professionals, as well as researchers have highlighted the importance of pain control while preserving the quality-of-life (QOL) of patients, the latter being recognized as one of the most important parameters to measure while assessing medical therapies. The guidelines also discussed the “importance of translating patient preferences for end-of-life care into specific physician medical orders” in regards to personalized medicine. In that context, opioids, and in particular morphine, remains the mainstay of analgesic therapy [1].

Unfortunately, pain management remains unsatisfactory and it is clear that different factors, including genetic and environmental factors, are important in identifying, designing, and targeting relevant interventions in different pain settings [2]. Traditionally, this variability has been explained by differences in bioavailability, metabolism, differences in pain perception, neurophysiological mechanisms, socio-cultural factors, as well as pharmacogenetic factors. Indeed, many studies have investigated the association between non-genetic/genetic factors and the variability of response to opioids in cancer patients [3]. Among genetic factors, polymorphisms, in particular single nucleotide polymorphisms (SNPs) in *OPRM1*, the gene encoding the mu opioid receptor and the most important target for morphine, are primary candidates for genetic influences on the efficacy of opioids. Numerous SNPs in the *OPRM1* have been identified, but only a few have been explored for possible relevance in opioid analgesia, including the c.118A>G SNP (rs1799971); patients carrying the GG genotype required higher morphine doses compared to AA patients [4,5]. Another relevant gene that has been identified as an important modulator of opioid efficacy and toxicity is the *COMT* gene encoding the catechol-*O*-methlytransferase. This enzyme metabolizes the catecholamines and is a key modulator of dopaminergic and adrenergic neurotransmission [6]. A common functional genetic variation in the *COMT* gene has been particularly studied: the c.472G>A polymorphism (rs4680; p.Val158Met) causes a valine (Val) to methionine (Met) substitution at codon 158 in the membrane-bound isoform enzyme, leading to a three- to four-fold reduced activity of the enzyme [7,8]. Patients with the Val allele have an enzyme up to four times more active than in patients with the Met allele and studies have shown that patients with the Met/Met genotype required lower morphine doses to achieve pain relief compared to Val/Met and Val/Val patients [6,9]. Finally, the c.3435C>T SNP (rs1045642) of *ABCB1* is an interesting factor affecting the pharmacokinetics of morphine. In fact, this gene encodes for P-glycoprotein (P-gp), a transmembrane efflux transporter that belongs to the family of ATP binding cassette (ABC) transporters and the variant results in a C-to-T substitution at nucleotide 3435 that has been associated with a reduced expression of duodenal P-gp in homozygous TT patients [10]. It has also been associated with a 1.5- to 2-fold reduction in mRNA levels and/or a reduction in protein expression in some tissues [11]. Publications have shown that *ABCB1* TT patients were “good responders” compared to CC patients [12] and required fewer morphine doses for pain relief [9,13,14].

Among all published studies, some heterogeneity in the inclusions could be noted as most of these studies included patients treated by various oral opioids (not a single molecule and rarely given intravenously) and from different pain settings (palliative and non-palliative settings). In our study, we decided to include only palliative care patients, especially patients in an end-of-life setting treated with intravenous morphine given via an infusion regimen. High pain scores and reduced QOL are two important dimensions that characterize these patients in the remaining days of their lives.

The main objective of this study was to investigate the association between genetic or non-genetic factors and morphine requirements in end-of-life setting cancer patients. The secondary objectives were to assess the relationship between these genetic and demographic/clinical factors on one side, and pain intensity and QOL on the other side. The ideal goal would be to increase the effectiveness of the available treatments, in particular morphine, and enhance the QOL of these patients.

## 2. Results

### 2.1. Patient Population

A total of 95 patients were enrolled in this study. Out of these patients, six were then excluded: five patients died before the scheduled date for blood sample collection and one did not consent to continue the evaluation due to his severe anxiety and depression.

Consequently, only 89 patients completed the study for the evaluation of pain management (Mean age 57 years old, 51.7% male and 48.3% female, 70% metastatic patients). Patients were treated by morphine for various types of cancer including gastrointestinal tract (*n* = 20; 22.5%), breast (*n* = 16; 18%), lung (*n* = 15; 16.9%), hematologic (*n* = 9; 10.1%), urogenital (*n* = 6; 6.7%), gynecologic (*n* = 6; 6.7%), prostate (*n* = 4; 4.5%), pancreas (*n* = 4; 4.5%), head and neck (*n* = 2; 2.25%), sarcoma (*n* = 2; 2.25%) and others (*n* = 5; 5.6%). The mean pain scores evaluated by the visual analog scale (VAS) at rest were 3.4 ± 2.93. These scores were not significantly different between the various types of cancer groups (*p* = 0.874; Table S1). Morphine doses, administered by continuous intravenous injection via pump infusion, were highly variable within this population: 7 to 210 mg per 24 h with mean doses of 34.78 ± 33.26 mg (Table 1). None of morphine doses nor the duration of morphine treatment differed significantly between the different types of cancer (Tables S2 and S3. The main clinical and demographic characteristics of patients are presented in Table 1.

### 2.2. Genotype and Allele Distribution

In order to determine the genetic background of the patients, genotype and allele frequencies of the studied SNPs were calculated. Results of the distribution as well as the comparison with other populations are summarized in Table 2. Concerning *OPRM1* c.118A>G, the allelic frequencies were 0.9 for 118A and 0.1 for 118G. For *COMT*, allelic frequencies were 0.51 for 158Val and 0.49 for 158Met and for *ABCB1* c.3435C>T, allelic frequencies were 0.54 for 3435C and 0.46 for 3435T (*n* = 168 chromosomes). The population was in Hardy–Weinberg equilibrium for the studied SNPs.

### 2.3. Variables Associated with Morphine Doses

A univariate analysis was conducted to explore the variables associated with morphine doses. The comparison of some socio-demographic and genetic characteristics regarding morphine dose at 24 h are presented in Table 3.

In addition, the morphine doses at 24 h were associated with age (*p* = 0.004) and the duration since the beginning of treatment with morphine (*p* < 0.001). However, no correlation was found between morphine doses and gender, weight, creatinine clearance, metastases, duration since diagnosis, and pain as evaluated by the VAS scores (*p* = 0.361, 0.997, 0.348, 0.476, 0.583, and 0.390, respectively). We also performed the Bonferroni correction for multiple testing; the only factor that remained significant was the duration since the beginning of treatment with morphine (*p =* 0.001 < *p*-value for Bonferroni Correction of 0.003).

The explanatory variables that showed associations with the doses of morphine with *p* < 0.25 in the univariate analyses (in bold in Table 3) were introduced in the multivariate model [19]. The results of the binary logistic regression for morphine dose at 24 h are presented in Table 4.

**Figure 1:**
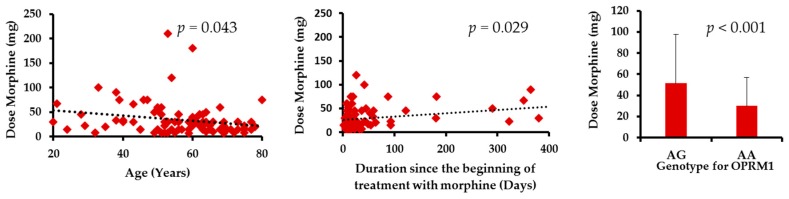
Representation of the variables associated with the morphine doses after multivariate analysis: the mean dose of morphine significantly decreased with age (*p* = 0.043) (**a**) but increased with the duration of morphine treatment (*p* = 0.029) (**b**); AG patients for the *OPRM1* c.118A>G SNP required significantly higher morphine doses than AA patients (*p* < 0.001) (**c**).

The regression analysis showed that age, duration since the beginning of morphine treatment, as well as *OPRM1* SNP were significantly associated with morphine doses at 24 h (*p* = 0.043, 0.029, and <0.001 respectively). The mean dose of morphine decreased with age but increased with the duration of morphine treatment. In addition, patients with at least one G allele for c.118A>G *OPRM1* (AG) needed higher morphine doses than AA patients (Figure 1).

### 2.4. Association to Pain

None of the studied factors (SNPs, gender, age, weight, creatinine clearance, dose of morphine, treatment duration, duration since cancer diagnosis, metastases, and concomitant use of other medications) were associated with the VAS scores at rest (Tables S4 and S5).

### 2.5. Variables Associated with Quality of Life and Morphine Side Effects

The patients’ QOL, as evaluated by the European Organisation for Research and Treatment of Cancer Core Quality of Life Questionnaire (EORTC-QLQ-C30 version 1.0) [20], including morphine side effects as well as the factors affecting it, were also assessed. Results are presented in Table 5.

To summarize, four factors were associated with the QOL in cancer patients: metastases, *OPRM1* SNP, age, and gender. Hence, our results have shown that the scores of the physical (PF) and role functioning (RF) as well as the scores for insomnia (SL) and global health status (GQOL) were significantly lower in patients with metastasis versus patients without metastasis (PF: 22.9 ± 29.39 versus 37.78 ± 37.76, *p* = 0.048; RF: 16.94 ± 33.81 versus 33.33 ± 43.85, *p* = 0.048; SL: 43.55 ± 42.07 versus 60.49 ± 42.4, *p* = 0.029, GQOL: 33.74 ± 26.67 versus 51.24 ± 29.57, *p* = 0.029). Moreover, AA patients for *OPRM1* SNP had significantly lower cognitive function than AG patients: 61.59 ± 32.38 versus 80 ± 28.41 (*p* = 0.014). In addition, age was associated with social functioning and financial difficulties: the social functioning scores increased with age but the financial difficulties scores decreased with age. Finally, women had significantly more nausea/vomiting than men with scores of 45.54 ± 42.51 versus 25.36 ± 32.72 (*p* = 0.016). None of these factors remained significant after Bonferroni correction in the univariate analysis.

## 3. Discussion

Pain management in patients with cancer, in particular in end-of-life settings, remains a challenge for clinicians due to unpredictable responses to opioid therapy. Pain has devastating consequences if unrelieved [21], causing numerous psychosocial responses [22,23] as well as severe and detrimental impacts on patients’ QOL [22,24]. A recent review of the literature determined the importance of overcoming barriers toward effective pain treatment and the need to develop and implement interventions to optimally manage pain in patients with cancer [22]. One of the interventions could be understanding the different factors affecting inter-individual variability in pain perception as well as drug efficacy and reported adverse drug reactions. Pharmacogenetics, also referred to as genotype-guided prescribing, is a new concept that aims to adapt medical treatments to patients’ genetic status [25]. It allows us to understand how the genetic variations could be used to tailor pain management therapies while improving the QOL of cancer patients. Recent guidelines highlighted the importance of assessing the risk of adverse effects of opioids used in pain treatment and outlined the precautions that help ensure that cancer patients with persistent pain use opioids safely and effectively [26].

In end-of-life pain settings, the use of infusion regimens for morphine delivery is indicated in patients in whom traditional administration routes are poorly effective or in those who cannot tolerate high doses because of systemic side effects [27,28,29]. However, the administrated morphine doses are highly variable and unpredictable among patients, who continue to experience intractable pain [27,28]. We therefore conducted this study to evaluate the added value of determining some genetic and non-genetic factors in optimizing cancer pain treatment, in particular in end-of-life settings.

In this study, we first evaluated the allelic frequency of *OPRM1* c.118A>G, *COMT* p.Val158Met, and *ABCB1* c.3435C>T. Allelic frequencies in our population were similar either to those described in Caucasian populations for *OPRM1* and *COMT* SNPs [16,17], or to those of Asian populations for *ABCB1* c.3435C>T [18]. This is not surprising since the Lebanese population is known for its admixture and numerous lineages [30]. As expected, the allelic distribution was similar to the one we previously reported in post-operative Lebanese patients (*n* = 95, [15]).

We then investigated the determinants of morphine doses, pain intensity, morphine side effects and the QOL of patients.

In our study, patient age and the duration of morphine therapy were significant predictors of morphine consumption. Elderly patients required significantly less morphine than younger patients. This could be due to altered pharmacokinetics of morphine in this population (distribution, metabolism, and elimination), which can explain their reduced need for morphine to achieve pain relief [31,32,33]. In addition, morphine doses increased with the duration of treatment as previously reported [9]. Two hypotheses could be put forward to explain this result. First, it is possible that this time interval is reflective of the progression of the disease; hence, they required higher morphine doses with the progression of their disease. Second, it could be due to the desensitization or down regulation of mu opioid receptors related to the repeated administration of morphine (tolerance) [34,35,36]. Moreover, patients with at least one 118G allele for *OPRM1* (AG, as none of the patients exhibited the GG genotype) received significantly higher doses of morphine than AA patients. Indeed, as previously described, the c.118A>G SNP in *OPRM1* induced a change from an asparagine to an aspartic acid residue at amino acid position 40 (p.N40D). This polymorphism is responsible for the loss of a putative N-linked glycosylation site in the N-terminal domain of the receptor, affecting the activation of the transduction pathway. In addition, the c.118A>G is associated with the μ opioid receptor (μOR) expression, the variant associated with a decrease in both mRNA expression and translation into a functional protein [37]. Studies have shown that the variant protein exhibits three times greater binding affinity for the endopeptide β-endorphin in vitro [38] and a reduced potency of morphine-6-glucuronide (M6G) [39], which explains the modified response to opiates [38]. This is probably why a homozygous carrier of the mutant 118G allele of *OPRM1* needs larger doses of morphine for pain control. Numerous studies have evaluated the association of *OPRM1* c.118A>G and the doses required for pain relief [3]. Results, such as those reported in the present study, have shown that patients carrying at least one 118G allele required higher morphine doses for cancer pain relief [4,5,9,12]. Nevertheless, the European Pharmacogenetic Opioid Study (EPOS), which included 2294 cancer patients, has not confirmed these results. The patients were however diagnosed by different types of cancer and were treated with various opioids (morphine, oxycodone, fentanyl, and other opioids) via numerous routes (oral, subcutaneous, intravenous, intrathecal, etc.) [40].

We did not detect any association between genetic or non-genetic factors with pain scores assessed by the VAS scores or the EORTC-QLQ-C30. Our results are in line with previously reported studies [9,41].

Concerning the QOL of our cancer patients, we identified different interesting factors significantly associated with various items of the EORTC-QLQ-C30 questionnaire. This latter is a very well recognized and validated assessment tool that has been formally translated into many languages. The main four factors were metastases, age, *OPRM1* SNP, and gender.

Hence, our results have shown that cancer patients with metastasis have lower physical and role functioning as well as a lower GQOL and a significantly higher incidence of insomnia. All these factors seem to be interrelated as patients with metastasis have many physical and psychosocial problems associated with the disease, its progression, and treatment that compromises their QOL [42]. In particular, patients with metastasis express more pain, anxiety, and depression [43,44] and many studies have suggested a bidirectional relationship between anxiety/depression and insomnia [45,46]. Moreover, insomnia is a major public health issue affecting the QOL of a large number of people all over the world with deleterious consequences such as long-term physical and mental exhaustion with altered mood, concentration, and memory. Therefore, people with insomnia have a deterioration of their general condition with a decrease in intellectual abilities and cognitive behavior [47,48].

Age was associated with social functioning and financial difficulties: the social functioning scores increased with age but the financial difficulties scores decreased with age. These results are accurate, as with age, the economic impact on the disease is less important than in younger patients and consequently, the financial difficulties are less pronounced. In addition, the impact of cancer, especially in end-of-life settings, on the social role of patients would be more problematic when the patient is young because of the increased prevalence of depression and anxiety [43].

Cognitive alterations in cancer patients are well described. They may be attributed to the cancer disease itself, comorbidities, and treatments including opioid therapy [49,50]. However, the effects of SNPs on the cognitive function of opioid-treated patients with cancer have rarely been explored. A recently published study aimed at identifying associations between 113 SNPs of 41 candidate genes, high opioid dose, and cognitive dysfunction [49]. Unfortunately, they did not explore SNPs in *OPRM1*. The association of the opioid system with cognitive function has been studied in particular with the dynorphin/κ-opioid receptor (κOR) but not with the mu-opioid receptor. Thus, studies have shown that the κOR system is implicated in emotion and cognition [51,52]. In addition, the pharmacological blockade of κOR prevented impairments in memory performance, whereas its activation induced cognitive deficits in mice [51,53]. In our study, AA patients for *OPRM1* SNP had significantly lower cognitive function than AG patients. To the best of our knowledge, this is the first study to report a significant association of the opioid system with the cognitive functioning of patients treated by morphine for pain. We could stipulate that patients with the AA genotype, exhibiting a better activation of the mu-receptor, would have cognitive deficits as described with the activation of the KOR, which can explain their lower cognitive function. In the future, it would be interesting to conduct more studies to better explore these patterns using validated tools for cognitive assessment such as the Mini-Mental State Examination (MMSE).

Furthermore, our study showed that women had significantly more nausea/vomiting than men, as previously described in a large sample of European patients treated by different opioids [54]. These results could be explained by the increased sensitivity of women treated by morphine due to hormonal variations.

Finally, our study showed that QOL was not significantly associated with morphine doses or with the type of cancer. However, the relatively small sample size in each category does not allow us to draw more conclusions.

### Strengths and Limitations

We recognize the strengths and limitations of this study. Our study included patients with a plethora of different cancers. We acknowledge that some types of cancers are related to more severe pain than others as the type of pain varies across cancer types; however, the VAS scores did not vary with the type of cancer as previously discussed. In addition, to the best of our knowledge, this is first study including a homogenous population of palliative care patients especially in an end-of-life setting, receiving a stable IV daily dose of morphine for a minimum of three consecutive days. These patients did not require any other opioid or intermittent morphine doses. We acknowledge as well that it is a relatively small study for genetic associations, but this study was conducted in a homogeneous sample of Lebanese patients and numerous previous studies had been conducted with a similar number of patients [55,56,57]. Further multi-centric studies with the same rigorous inclusion criteria are consequently needed on a larger sample to confirm and generalize our results on Lebanese patients, as well as for other populations. It would be interesting to include patients treated by a single molecule (morphine, oxycodone, fentanyl, etc.) and diagnosed with a single type of cancer to reduce the heterogeneity of the included population. As for the clinical assessment (for cancer pain and quality of life), researchers should consider using modular approaches for disease-specific treatment measurements such as the European Organization for Research and Treatment-QOL questionnaire EORTC-QLQ-BR23 or the functional assessment of cancer-therapy breast QOL (FACT-B) for breast cancer, the EORTC-QLQ-BN20 or FACT-Br for primary brain tumors, the EORTC-QLQ-CR29 FACT-C for colorectal cancer, etc. [58,59,60]. In fact, even if the EORTC-QLQ-C30 is a well-validated assessment tool, it remains a general tool assessing the generic aspects of QOL and does not take into account the symptoms related to a specific tumour site or the treatment modality (side effects associated with a given treatment) or additional QOL domains affected by the disease or treatment (e.g., sexuality, body-image, fear of disease recurrence, etc.) [58,59,60]. A final point that should be raised within this study is the chance of false positives in multiple testing. In fact, when different factors are assessed with specific endpoints (such as morphine doses or QOL items in our study), repeated statistical tests on the same data will cause α inflation and lead the researcher to a higher probability of making a Type I error. Therefore, it would be much more likely to report significant differences between some of the pairs that have no real difference. To overcome this problem, some corrections for multiple testing could be performed. In our study, although we performed multivariate analysis, we included the results of the Bonferroni correction for its information. The only factor that remained significant after the correction was the duration since the beginning of the treatment with morphine, which drives us to be more cautious when interpreting the results.

## 4. Materials and Methods

### 4.1. Study Design and Patients

Patients admitted to the Department of Hemato-oncology at Hôtel-Dieu de France Hospital (Saint-Joseph University of Beirut, Beirut, Lebanon) were enrolled in this prospective study from 14 January 2011 until 30 November 2016. The study was approved by the hospital ethical committee (Protocol No. 336, 2013) and all patients gave their written informed consent.

All included patients were above 18 years old, were diagnosed as suffering from malignant diseases as described in the results section, and had received scheduled morphine treatment corresponding to step III at the analgesic ladder of the World Health Organization (WHO) [61]. Patients were treated with a stable morphine dose for at least three days before inclusion (no changes in the morphine dose occurred during the last three days).

Patients received an initial morphine dose via infusion pump followed by a titration until VAS < 4 (morphine diluted in normal saline serum; infusion rate 0.5 mL/h over 24 h). If the VAS ≥ 4, rescue doses of morphine (corresponding to 1/6 of the baseline daily dose) were administered. The total 24 h dose as well as pain scores were reevaluated every 24 or 48 h: if the patient required four or more rescue doses of morphine during 24 h, a new baseline dose was calculated. The new baseline dose was the sum of the old baseline and the total rescue doses needed. This baseline dose was maintained until the VAS score became ≥4, and then consequently new rescue doses were recalculated.

Other opioids from the step III analgesic ladder were not allowed. Only oral tramadol or codeine were permitted on demand if the patient still felt pain. It is noteworthy to add that none of the patients received or had received chemo or radiotherapy in the previous two weeks before enrollment.

Kidney function was assessed prior to enrollment by estimated creatinine clearance e-Clcr (using Cockcroft-Gault formula) and estimated Glomerular Filtration Rate e-GFR (using Modification of Diet in Renal Disease MDRD formula). Patients with e-Clcr less than 25 mL/min or e-GFR less than 25 mL/min/1.73 m^2^ were not included in the study.

Clinical and demographic information including age, gender, weight, height for the body mass index (BMI) calculation, ethnicity, time since the start of morphine treatment, cancer diagnosis and localization of metastases, and co-medication were registered by a health care provider (physician or pharmacist). Patients’ quality of life was evaluated and reported by answering the EORTC-QLQ-C30. This questionnaire is a very well recognized and validated assessment tool that has been formally translated into many languages. Moreover, all evaluated items of the EORTC-QLQ-C30 are independent [20].

Hence, the functional state of the patients, including “Physical functioning” and “Role functioning” during the previous week, was assessed by answering a “yes” or “no” for the asked questions.

For the rest of the items in the functional state (social functioning, emotional functioning, cognitive functioning) and for all the items evaluating symptom intensity during the previous week, a four-point verbal rating scale with the notations “not at all, a little, quite a bit, and very much” was used. Social, emotional, and cognitive functioning were assessed using scores calculated by the answers of combined items of the EORTC questionnaire (items 26–27, items 21–24, items 20–25 respectively; Table S6) as recommended [20].

Reported symptoms were: fatigue, pain, nausea/vomiting, dyspnea, insomnia, loss of appetite, constipation, and diarrhea. In addition, and apart from the EORTC-QLQ-C30 pain score, pain was self-rated by the patients using the item of “average pain” by the VAS during a 24 h period. Briefly, patients rated pain on a numeric scale, where 0 represents “no pain” and 10 represents “pain as bad as you can imagine”, as recommended for use in clinical studies of pain. The pain scores were reported in integral numbers. In order to avoid confusion in the interpretation of results, the pain rated by the EORTC-30 score will be noted as the “normalized pain score” and the one evaluated by the VAS score will be mentioned as the “VAS score”.

Finally, patients were able to relate their global health status/quality of life. This dimension employed the 7-point response scale, from 1 to 7, with “1” corresponding to a very poor quality of life and “7” to an excellent quality of life.

### 4.2. Genotyping

DNA was extracted from blood cells using the QIAamp DNA Mini^®^ Blood (Qiamp DNA Mini kit cat nb: 51304, QIAGEN^®^, Hilden, Germany), as recommended by the manufacturer.

Genotyping for the three SNPs was performed using the Lightcycler^®^ 2.0 (Roche Diagnostics GmbH, Mannheim, Germany).

In summary, the reaction was carried out using 25 ng of DNA (10 ng/μL solution or 2.5 µL) in a final volume of 10 µL. The reaction mixture (10 μL) contained Fast Start Taq polymerase (10×), buffer and dNTPs, MgCl_2_ (10 mM); Lightcycler Fast Start DNA Master Hybridization Probes Kit^®^ (catalogue no. 03 003 248 001, Roche Diagnostics GmbH), and 0.2 μL of each primer (20 mM) and fluorescent probes (anchor and sensor, 20 mM) (TIB Molbiol^®^, TIBMOLBIOL, Berlin, Germany). The samples were then loaded into composite plastic/glass capillaries (20 μL LC capillaries, Roche Diagnosis, catalogue no. 04 929 292 001, Roche Diagnostics GmbH), centrifuged, and were placed in the LightCycler sample carousel.

Genotyping of *OPRM1* (rs1799971) and *ABCB1* (rs1045642) were performed according to previously published methods [62,63]. Primers and probes were synthesized using TIB MOLBIOL Syntheselabor GmbH, Berlin, Germany. The sequences were as follows: for *OPRM1* c.118A>G, primer forward 5′-GCTTGGAACCCGAAAAGT-3′, primer reverse 5′-GTAGAGGGCCATGATCGTGA-3′, probes CCCGGTTCCTGGGTCAACTTGTCC-FL and 640-CTTAGATGGCAACCTGTCCGACC-PH and for ABCB1 c.3435C>T, primer forward 5′-TGTTTTCAGCTGCTTGATGG-3′, primer reverse 5′-AAGGCATGTATGTTGGCCTC-3′, probes 640-GACAACAGCCGGGTGGTGTCA and GGAAGAGATCGTGAGGGCAG-PH. For *COMT*, primers were selected using the Primer 3 software [64] and were synthesized by TIB MOLBIOL Syntheselabor GmbH, Berlin, Germany.

The PCR protocol and conditions are presented in the Table S7. Positive heterozygous and homozygous controls (defined by direct sequencing) and negative controls (water) were systematically included in experiments.

The genotyping was conducted on patients following their evaluation. The genotyping was performed in the laboratory and none of the investigators, clinical care providers, or observers of this study were aware of the genotyping results. Therefore, the genetic testing could not have biased the pain assessment process.

### 4.3. Data and Statistical Analysis

Clinical data are presented as mean ± standard deviation (SD). The statistical analysis was performed using a software program (Statistical Package Software for Social Science—SPSS—for Windows version 16.0, SPSS Inc., Chicago, IL, USA). The α error was set at 0.05.

The formula for calculating the sample size requirements was the one published by Tabachnick and Fidell [65] that takes into account the number of independent variables included in the model: *n* = 50 + 8 *m* (*m* is the number of independent variables); given that *m* = 5, at least 90 subjects have to be included in the present study.

Deviation from the Hardy–Weinberg equilibrium was tested using χ^2^ analysis with one degree of freedom.

To determine the genetic and non-genetic factors associated with the morphine doses at 24 h, univariate analyses of categorical and continuous variables were carried out using the Mann–Whitney test or Kruskal–Wallis test and the Spearman correlation coefficient, respectively. Multiple regression analysis was used with the dose of morphine as the dependent continuous variable. Variables that showed associations with *p*-values < 0.25 in univariate analyses were candidates for the multivariate model, according to the Enter method [19]. Collinearity among the independent variables was also tested. Independent variables that highly correlated were excluded. It has already been suggested not to include two independent variables where there is a correlation of 0.7 or more. Multiple regression analyses were also used for each of the quality of life domain as the dependent variable.

Finally, we included the Bonferroni Correction for univariate analysis (pairwise comparisons using the Dunn-Bonferroni approach) that provides more confidence in the results because it reduces the probability of a Type I error by its limits on α inflation in multiple testing. Considering that morphine doses were assessed with 14 factors, we needed a *p*-value < 0.003 for statistical significance. Moreover, each item of the QOL was assessed with 9 factors, and the significant *p*-value was therefore set to a value <0.005.

## Figures and Tables

**Table 1:** Characteristics of the patients.

Characteristics of the Subjects (*n* = 89)	*n* (%)
Men	46 (51.7)
Women	43 (48.3)
Ethnicity	
Lebanese	84 (94.4)
Non-Lebanese ^1^	5 (5.6)
Diagnosis	
Gastrointestinal tract	20 (22.5)
Breast	16 (18.0)
Lung	15 (16.9)
Hematologic	9 (10.1)
Urogenital	6 (6.7)
Gynecologic	6 (6.7)
Prostate	4 (4.5)
Pancreas	4 (4.5)
Head and neck	2 (2.25)
Sarcoma	2 (2.25)
Others	5 (5.6)
Metastases	62 (69.7)
Concomitant use of opioids ^2^	6 (6.7)
Concomitant use of other drugs for neuropathic pain ^3^	64 (71.9)
**Variables**	**Mean ± SD**
Age (years)	56.94 ± 13.88
Weight (kg)	64.41 ± 14.26
Creatinine clearance (mL/min)	121.01 ± 46.68
Visual analog scale (VAS) score at rest at 24 h	3.4 ± 2.93
Dose of morphine at 24 h (mean ± SD—mg) Pump infusion	34.78 ± 33.26
Duration of morphine treatment (mean ± SD—days)	121.96 ± 383.87
Duration since cancer diagnosis (mean ± SD—months)	37.08 ± 50.65

^1^—Non-Lebanese included one patient from Syria, three from Iraq, and one from France. These patients were only excluded from the allelic frequencies calculation but included in the evaluation of the factors associated with morphine doses and quality of life QOL; ^2^—The concomitant use of opioids includes only oral tramadol or codeine administered to the patient on demand if the patient still feels pain; no other opioids were permitted, otherwise the patient was not included in the study; ^3^—The concomitant use of other drugs for neuropathic pain includes drugs taken by the patient during the previous week for this indication. In our population, the only reported drugs were clomipramine, duloxetine, gabapentin, and pregabalin.

**Table 2:** Genotype and allele frequencies of *OPRM1*, *COMT*, and *ABCB1* variants in our population. Comparison with previously published data.

Gene dbSNP	Genotype Frequencies ^1^			Allelic Frequencies		*p* ^2^
*OPRM1* rs1799971	AA	AG	GG	A	G	
Lebanese patients *n* = 84 ^3^ (Current study, cancer)	67 (79.8)	17 (20.2)	0 (0.0)	0.90	0.10	–
Lebanese patients *n* = 96 [15]	76 (79.2)	18 (18.8)	2 (2.1)	0.89	0.11	0.41
European HapMap *n* = 113	80 (70.8)	31 (27.4)	2 (1.8)	0.84	0.16	0.22
Japanese HapMap *n* = 86	29 (33.7)	34 (39.5)	23 (26.7)	0.53	0.47	<0.0001 *
Chinese HapMap *n* = 43	18 (41.9)	19 (44.2)	6 (14)	0.64	0.36	<0.0001 *
Sub-Saharan African HapMap *n* = 60	60 (100)	0 (0)	0 (0)	1	0	0.001 *
*COMT* rs4680	Val/Val	Val/Met	Met/Met	Val	Met	
Lebanese patients *n* = 84 ^3^ (Current study, cancer)	22 (26.2)	42 (50)	20 (23.8)	0.51	0.49	–
Lebanese patients *n* = 96 [15]	23 (24)	48 (50)	25 (26)	0.49	0.51	0.92
European HapMap *n* = 113	28 (24.8)	52 (46)	33 (29.2)	0.48	0.52	0.67
Japanese HapMap *n* = 86	6 (7)	37 (43)	43 (50)	0.28	0.72	<0.0001 *
Chinese HapMap *n* = 43	3 (7)	14 (32.5)	26 (60.5)	0.23	0.77	0.0001 *
Sub-Saharan African HapMap *n* = 113	10 (8.8)	51 (45.2)	52 (46)	0.31	0.69	0.0004 *
*ABCB1* rs1045642	CC	CT	TT	C	T	
Lebanese patients *n* = 84 ^3^ (Current study, cancer)	29 (34.5)	32 (38.1)	23 (27.4)	0.54	0.46	–
Lebanese patients *n* = 96 [15]	34 (35.4)	38 (39.6)	24 (25)	0.55	0.44	0.94
European HapMap *n* = 113	17 (15)	63 (55.8)	33 (29.2)	0.43	0.57	0.0041 *
Japanese HapMap *n* = 86	22 (25.6)	49 (57)	15 (17.4)	0.54	0.46	0.045 *
Chinese HapMap *n* = 42	16 (38.1)	17 (40.5)	9 (21.4)	0.58	0.42	0.76
Sub-Saharan African HapMap *n* = 113	89 (78.8)	23 (20.4)	1 (0.8)	0.89	0.11	<0.0001 *

^1^—Value represents the number of patients with the percentage shown in parenthesis; ^2^—*p*-Values are obtained using the χ^2^ test between the number of patients of each genotype compared to our study [16,17,18]; ^3^—The total number of patients included in this table is only 84, as non-Lebanese were excluded from this analysis. * Statistically significant result.

**Table 3:** Comparisons of some socio-demographic and genetic characteristics between the groups of subjects differing in morphine dose at 24 h.

Characteristics of the Subjects	*n*	Dose of Morphine at 24 h (Mean—mg)	Standard Deviation	*p*
***OPRM1* c.118A>G**				
**AA**	**69**	**29.97**	**26.96**	**0.010 ^2^**
**AG ^1^**	**20**	**51.37**	**46.35**	
*COMT p.Val158Met*				
Val/Val	23	34.85	26.96	0.307
Val/Met	43	30.52	33.06	
Met/Met	23	42.68	38.87	
Val/Val	23	34.85	26.96	0.701
ValMet/MetMet	66	34.76	35.38	
*ABCB1* c.3435C>T				
CC	29	32.87	22.75	0.857
CT	35	41.38	46.41	
CC	25	27.76	16.75	
CC	29	32.87	22.75	0.750
CT/TT	60	35.71	37.44	
Metastasis				
Yes	62	32.54	28.92	0.476
No	27	39.94	41.73	
**Concomitant use of opioids ^3^**				
**Yes**	**6**	**22.50**	**8.22**	**0.015**
**No**	**83**	**35.67**	**34.22**	
**Concomitant use of other drugs** **^4^**				
**Yes**	**64**	**39.63**	**37.37**	**0.002**
**No**	**25**	**22.36**	**12.94**	

^1^—No GG patients were identified in our study; ^2^—The variables highlighted in bold represent the explanatory variables that showed associations to the doses of morphine with *p* < 0.25 in the univariate analyses; ^3^—The concomitant use of opioids includes only oral tramadol or codeine administered to the patient on demand if the patient still feels pain; no other opioids were permitted, otherwise the patient was not included in the study; ^4^—The concomitant use of other drugs for neuropathic pain includes drugs taken by the patient during the previous week for this indication. In our population, the only reported drugs were clomipramine, duloxetine, gabapentin, and pregabalin.

**Table 4:** Binary logistic regression for morphine dose at 24 h.

Doses de Morphine	Unstandardized Coefficients		Standardized Coefficients	*t* ^4^	Sig. ^5^	95% Confidence Interval (CI) for B	
	B ^1^	S.E. ^2^	β ^3^			Lower Bound	Upper Bound
**Age ^6^**	**−0.342**	**0.167**	**−0.143**	**−2.053**	**0.043**	**−0.675**	**−0.010**
**Treatment duration**	**0.061**	**0.006**	**0.700**	**10.112**	**<0.001**	**0.049**	**0.073**
***OPRM1* c.118A>G**	**12.838**	**5.756**	**0.162**	**2.230**	**0.029**	**1.381**	**24.295**
Concomitant use of other drugs	−10.525	6.567	−0.119	−1.603	0.113	−23.596	2.547
Concomitant opioids use	2.440	9.096	0.018	0.268	0.789	−15.665	20.545

This is a table of multivariate analyses where all the confounding factors were included in the model in order to study the adjusted association of each explanatory independent variable with the dose of morphine. ^1^—B is the unstandardized regression coefficient; ^2^—S.E. is the standard error; ^3^—β is the standardized regression coefficient; ^4^—*t* is the *t*-test; ^5^—Sig. is the significance level or the *p*-value; ^6^—The variables highlighted in bold represent the explanatory variables that showed associations to the doses of morphine with *p* < 0.05 in the multivariate analyses.

**Table 5:** Variables associated with the QOL in univariate and multivariate analysis.

Items of the QOL ^1^	*n*	Mean	Std. Deviation	Univariate Analyses	*p* ^2^	Multivariate Analyses	Std. Error ^4^	Standardized Coefficients	95% CI for B ^6^	Upper Bound	*p* ^7^
						Unstandardized Coefficients B ^3^		β ^5^	Lower Bound		
**Physical functioning (PF)**	89	27.42	32.67	**Metastases ^8^**	**0.048**	-	-	-	-	-	-
**Role functioning (RF)**	89	21.91	37.66	*ABCB1* SNP	0.159	−9.185	8.898	−0.115	−26.883	8.512	0.305
				*OPRM1* SNP	0.119	10.335	9.676	0.115	−8.910	29.579	0.289
				**Metastases**	0.059	−8.745	7.888	−0.117	0.126	35.609	**0.048**
				Gender	0.189	17.867	8.92	0.219	-0.513	0.709	0.751
**Social functioning (SF)**	89	52.62	38.43	*OPRM1* SNP	0.097	15.961	9.444	0.174	−2.812	34.734	0.095
				**Age**	0.034	0.646	0.286	0.233	0.078	1.213	**0.026**
Emotional functioning (EF)	89	44.8	34.36	*ABCB1* SNP	0.191	−9.805	7.741	−0.123	−25.193	5.583	0.209
				Gender	0.18	−7.695	7.26	−0.113	−22.128	6.738	0.292
**Cognitive functioning (CF)**	89	65.73	32.31	***OPRM1* SNP**	0.014	19.448	7.985	0.253	3.571	35.325	**0.017**
				*COMT* SNP	0.195	−9.019	7.706	−0.123	−24.340	6.303	0.245
				Metastases	0.209	8.109	7.343	0.116	−6.491	22.709	0.273
Fatigue (FA)	89	74.28	26.04	*COMT* SNP	0.156	−10.142	6.197	−0.171	−22.461	2.176	0.105
				Gender	0.055	10.624	5.429	0.205	-0.167	21.416	0.054
Pain (PA) (Normalized Pain Scores)	89	70.32	32.86	Age	0.189	−0.286	0.259	−0.121	−0.801	0.23	0.273
				Gender	0.189	8.85	6.888	0.135	−4.845	22.545	0.202
				Metastases	0.104	8.824	7.781	0.124	−6.647	24.295	0.26
**Nausea and vomiting (NV)**	89	35.11	38.89	*OPRM1* SNP	0.087	−9.604	9.964	−0.104	−29.419	10.21	0.338
				**Gender**	0.038	19.578	7.969	0.253	3.731	35.426	**0.016**
				Morphine dose	0.132	−0.177	0.125	−0.151	−0.425	0.071	0.16
				VAS scores ^9^		1.481	1.37	0.111	−1.242	4.205	0.282
**Global health status/QoL (GQOL)**	89	39.05	28.58	*COMT* SNP	0.193	−8.297	6.75	−0.128	−21.715	5.122	0.222
				**Metastases**	0.007	16.185	6.428	0.262	3.406	28.963	**0.014**
Dyspnea (DY)	89	32.4	33.93	-							
**Insomnia (SL)**	89	48.69	42.66	*ABCB1* SNP	0.098	−11.636	9.66	−0.129	−30.842	7.571	0.232
				**Metastases**	0.095	22.028	9.898	0.239	2.348	41.708	**0.029**
				Age	0.216	0.604	0.341	0.196	−0.075	1.282	0.08
Appetite loss (AP)	89	68.91	39.5	-							
Constipation (CO)	89	60.67	42.51	-							
Diarrhea (DI)	89	12.73	26.83	-							
**Financial difficulties (FI)**	89	35.21	33.84	*ABCB1* SNP	0.234	3.887	7.628	0.054	−11.277	19.051	0.612
				**Age**	0.004	−0.699	0.259	−0.287	−1.214	-0.184	**0.008**

^1^—All items of the European Organisation for Research and Treatment of Cancer Core Quality of Life Questionnaire (EORTC-QLQ-C30) are independent [20]; ^2^—The explanatory variables that showed associations with the doses of morphine with *p* < 0.25 in the univariate analyses were introduced in the multivariate model; ^3^—B is the unstandardized regression coefficient; ^4^—Std. Error is the standard error; ^5^—β is the standardized regression coefficient; ^6^—CI is Confidence Interval for B; ^7^—*p*-Values for the multivariate analyses; ^8^—The variables highlighted in bold represent the explanatory variables that showed associations with the doses of morphine with *p* < 0.05 in the multivariate analyses; ^9^—We decided to include pain as evaluated by the visual analog scale (VAS) scores in the multivariate analysis of the nausea/vomiting because we thought that these symptoms could be affected by the intensity of pain and we did not want it to bias the rest of the variables.

## Supplementary Materials

Supplementary materials can be found at www.mdpi.com/1422-0067/18/4/669/s1.

## Abbreviations

*ABCB1*	ATP-binding cassette, subfamily B, member 1 gene
AP	Appetite loss
ASCO	American Society of Clinical Oncology
BMI	Body mass index
CF	Cognitive functioning
CI	Confidence Interval
CO	Constipation
COMT	Catechol-*O*-methyltransferase (enzyme and gene)
DI	Diarrhea
DNA	Deoxyribonucleic acid
DY	Dyspnea
EDTA	Ethylenediaminetetraacetic acid
EF	Emotional functioning
EORTC-QLQ-C30	European Organisation for Research and Treatment of Cancer Core Quality of Life Questionnaire
EPOS	European Pharmacogenetic Opioid Study
FA	Fatigue
FI	Financial difficulties
GQOL	Global health status/QoL
MMSE	Mini-Mental State Examination
NV	Nausea and vomiting
PA	Pain
*OPRM1*	Mu opioid receptor gene
PF	Physical functioning
P-gp	P-glycoprotein
RF	Role functioning
SD	Standard deviation
SF	Social functioning
SL	Insomnia
QOL	Quality-of-life
SNP	Single nucleotide polymorphism
SPSS	Statistical Package Software for Social Science (for Windows)
VAS	Visual analog scale
WHO	World Health Organization
