# Supplementary material for: OPRM1 c.118A>G Polymorphism and Duration of Morphine Treatment Associated with Morphine Doses and Quality-of-Life in Palliative Cancer Pain Settings
Source: Int J Mol Sci. 2017 Mar 27;18(4):669. doi: 10.3390/ijms18040669 (PMC5412267; doi:10.3390/ijms18040669)
Supplement: Supplementary file 1 [file ijms-18-00669-s001.pdf]

Table S1. Association between VAS scores and various types of cancer groups.

		N	Mean	Std. Deviation	*p*
VAS scores	Urogenital	6	3.33	3.011	0.874
	Lung	15	2.73	2.520	
	Breast	16	3.31	3.219	
	Prostate	4	3.38	3.902	
	Gastrointestinal tract	20	4.58	3.006	
	Gynecologic	6	2.67	3.204	
	Hematologic	9	3.67	3.240	
	Sarcoma	2	3.50	4.950	
	Head and neck	2	2.50	3.536	
	Pancreas	4	2.25	2.062	
	Others	5	2.80	1.924	

Table S2. Association between morphine doses and various types of cancer groups.

		N	Mean	Std. Deviation	*p*
Morphine Doses	Urogenital	6	26.6667	14.11264	0.129
	Lung	15	33.1667	28.16512	
	Breast	16	44.6875	41.56195	
	Prostate	4	20.4000	6.73102	
	Gastrointestinal tract	20	30.4980	17.35949	
	Gynecologic	6	22.3600	26.07983	
	Hematologic	9	36.2000	27.94231	
	Sarcoma	2	83.3000	23.61737	
	Head and neck	2	18.7500	15.90990	
	Pancreas	4	73.1250	92.54222	
	Others	5	15.0000	5.30330	

Table S3. Association between treatment duration and various types of cancer groups.

		N	Mean	Std. Deviation	*p*
Treatment duration	Urogenital	6	75.33	122.469	0.111
	Lung	13	16.23	14.805	
	Breast	16	240.12	571.990	
	Prostate	4	32.50	27.923	
	Gastrointestinal tract	20	77.90	171.702	
	Gynecologic	5	30.80	34.215	
	Hematologic	9	100.33	169.801	
	Sarcoma	2	196.00	219.203	
	Head and neck	2	7.00	2.828	
	Pancreas	3	866.33	1465.108	
	Others	5	22.40	39.532	

Table S4. Association between VAS scores and different demographic and genetic factors.

		N	Mean VAS Scores	Std. Deviation	*p*
**Sexe**					0.960
	Men	46	3.34	2.864	
	Women	43	3.48	3.026	
***COMT* SNP**					0.541
	ValMet/MetMet	66	3.27	2.924	
	ValVal	23	3.78	2.969	
***ABCB1* SNP**					0.664
	CT/TT	60	3.30	2.860	
	CC	29	3.62	3.101	
***OPRM1* SNP**					0.224
	AA	69	3.61	2.948	
	AG	20	2.70	2.812	
**Metastasis**					0.476
	Oui	62	3.37	3.049	
	Non	27	3.48	2.680	
**Concomitant opioids use**					0.218
	Oui	6	4.83	2.229	
	Non	83	3.30	2.955	
**Concomitant use of other drugs**					0.388
	Oui	64	3.59	3.070	
	Non	25	2.92	2.515	

Table S5. Association between VAS scores and age, weight, creatinine clearance, treatment duration, morphine dose and duration since the beginning of treatment.

		VAS scores
Age	Correlation Coefficient	-.125
	-p-value	.242
	N	89
Weight	Correlation Coefficient	.058
	Sig. (2-tailed)	.590
	N	89
Creatinine clearance	Correlation Coefficient	-.100
	Sig. (2-tailed)	.352
	N	88
Treatment duration	Correlation Coefficient	.-.052
	Sig. (2-tailed)	.635
	N	85
Morphine dose	Correlation Coefficient	.092
	Sig. (2-tailed)	.390
	N	89
Duration since diagnosis	Correlation Coefficient	-.047
	Sig. (2-tailed)	.669
	N	85

Table S6. The PCR protocol and conditions for *OPRM1*, *COMT* and *ABCB1* genotyping.

	**PCR**				**FUSION CURVES**	
OPRM1 c.118A>G	5 s à 95°C	10 s à 55°C	15 s à 72°C	45 cycles	95 s à 40°C	De 40°C à 85°C à 0,2°C/s
COMT p.Val158Met	5 s à 95°C	10 s à 60°C	15 s à 72°C	45 cycles	95 s à 40°C	De 40°C à 85°C à 0,2°C/s
ABCB1 c.3435C>T	5 s à 95°C	10 s à 54°C	15 s à 72°C	45 cycles	95 s à 40°C	De 40°C à 85°C à 0,2°C/s

Table S7. The EORTC-QLQ-C30 version 1.0 with functional / symptom scales indicated.

	SCALE	NO	YES
1. Do you have any trouble doing strenuous activities, like carrying a heavy shopping bag or a suitcase?	Physical	1	2
2. Do you have any trouble taking a **long** walk?	Physical	1	2
3. Do you have any trouble take a **short** walk outside of the house?	Physical	1	2
4. Do have to stay in bed or a chair for most of the day?	Physical	1	2
5. Do you need help with eating, dressing, washing yourself or using the toilet?	Physical	1	2
6. Are you limited in any way in doing either your work or doing household jobs?	Role	1	2
7. Are you completely unable to work at a job or to do household jobs?	Role	1	2

**During the past week:**	SCALE	**Not at all**	**A little**	**Quite a bit**	**Very much**
8. Were you short of breath?	Dyspnoea	1	2	3	4
9. Have you had pain?	Pain	1	2	3	4
10. Did you need rest?	Fatigue	1	2	3	4
11. Have you had trouble sleeping?	Insomnia	1	2	3	4
12. Have you felt weak?	Fatigue	1	2	3	4
13. Have you lacked appetite?	Appetite Loss	1	2	3	4
14. Have you felt nauseated?	Nausea and Vomiting	1	2	3	4
15. Have you vomited?	Nausea and Vomiting	1	2	3	4

**During the past week:**	SCALE	**Not at all**	**A little**	**Quite a bit**	**Very much**
16. Have you been constipated?	Constipation	1	2	3	4
17. Have you had diarrhoea?	Diarrhoea	1	2	3	4
18. Were you tired?	Fatigue	1	2	3	4
19. Did pain interfere with you daily activities?	Pain	1	2	3	4
20. Have you had difficulty in concentrating on things, like reading a newspaper or watching television?	Cognitive	1	2	3	4
21. Did you feel tense?	Emotional	1	2	3	4
22. Did you worry?	Emotional	1	2	3	4
23. Did you feel irritable?	Emotional	1	2	3	4
24. Did you feel depressed?	Emotional	1	2	3	4
25. Have you had difficulty remembering things?	Cognitive	1	2	3	4
26. Has your physical condition or medical treatment interfered with your **family** life?	Social	1	2	3	4
27. Has your physical condition or medical treatment interfered with your **social** activities?	Social	1	2	3	4
28. Has your physical condition or medical treatment caused you financial difficulties?	Financial Difficulties	1	2	3	4

GLOBAL HEALTH STATUS						
29. How would you rate your overall **physical condition** during the past week?						
1 Very poor	2	3	4	5	6	7 Excellent
30. How would you rate your overall **quality of life** during the past week?						
1 Very poor	2	3	4	5	6	7 Excellent
